# Supplementary material for: Trail Management Terminology and Decision-Making: A Conceptual and Practical Framework
Source: Environ Manage. 2026 Feb 25;76(4):117. doi: 10.1007/s00267-026-02394-4 (PMC12935811; doi:10.1007/s00267-026-02394-4)
Supplement: Supplementary file 1 — Supplementary information [file 267_2026_2394_MOESM1_ESM.docx]

**Trail Management Terminology and Decision-Making: A Conceptual and Practical Framework**

Marcos Vinícius Ribeiro de Castro Simão ^a, b,c,^ *; Manel Llena ^b^, Estela Inés Farías-Torbidoni ^c^,

^a^ Federal Institute of Education, Science and Technology of Amazonas - *campus* Tefé - IFAM, Brazil

^b^ Fluvial Dynamics Research Group (RIUS), University of Lleida (UdL), Lleida, Spain

^c^ National Institute of Physical Education of Catalonia (INEFC) - University of Lleida (UdL), Lleida, Spain

* Marcos Vinícius Ribeiro de Castro Simão ([mrd14@alumnes.udl.cat](mailto:mrd14@alumnes.udl.cat))

Orcid numbers: Marcos Vinícius Ribeiro de Castro Simão (0000-0002-8738-6350); Manel Llena (0000-0001-7095-6188); Estela Inés Farías-Torbidoni (0000-0002-7541-8140)

**Abstract**

Trail management in protected natural areas seeks to maximize opportunities for outdoor recreation and the provision of cultural ecosystem services while minimizing deleterious changes to trail-associated natural resources. The field’s interdisciplinarity, drawing on recreation ecology, trail science, and restoration ecology, enriches the knowledge base and practice of trail management, yet it can also lead to inconsistent use and application of cross-disciplinary terminology. We conducted a systematic literature review with summative content analysis to examine four term sets: (i) terms for deleterious physical changes affecting trail usability (impact, damage, degradation), (ii) terms for the creation of new trails (construction, build), (iii) terms for the care of existing trails (maintenance, repair), and (iv) terms for reversing trail degradation (rehabilitation, restoration, renaturalization, recovery), whether to return a trail to functional use or to advance ecological restoration toward a designated reference. Based on this analysis, we introduce two conceptual outputs that organize terminology and map relationships among terms. Finally, to translate these outputs into practice, we present a decision-making flowchart to support managers in selecting trail interventions aligned with explicit management goals. Clarifying overlaps and transitions among principal terms can improve communication among stakeholders, help identify ecological thresholds, and guide timely choices between sustaining functional trail use and shifting toward strategies that emphasize biodiversity conservation.

**Keywords** Trail management decision-making • Ecological restoration • Trail management terminology • Trail degradation

# Introduction

Recreational trails in natural areas occur across diverse ecosystems, climates and topographies, and are governed by heterogeneous societal, cultural, and legal frameworks. Consequently, outdoor recreation and trail management are complex endeavors that draw on knowledge from multiple scientific fields (Marion 2016), including conservation ecology, recreation ecology and restoration ecology. Management of recreational activities in natural areas, and along their trails, relies primarily on evidence generated in recreation ecology, which investigates the impacts of outdoor recreation on natural resources and approaches for managing those impacts (e.g. Coleman 1977; Hammitt et al. 2015; Salesa and Cerdà 2020; Smith and Pickering 2024; Llena 2025). Within this broader field, the management of recreational trails, often referred to as trail science and considered a branch of recreation ecology, likewise integrates diverse knowledge to inform decision-making (Marion 2023).

This broad diversity of disciplines on which trail management can draw clearly enriches trail science, making it more robust and better grounded to address challenges arising from the dynamics of outdoor recreation (e. g. Tomczyk et al. 2016). Conversely, communication across disciplines can be difficult because each relies on specialized technical terminology. At times, different fields use the same term with very similar definitions; at other times, a single term denotes distinct practices or actions, and in still other cases, different activities are labeled with the same term. Even within a single field, the definition of a term may change as scientific understanding advances over time. Such shifts can involve core terms, and misinterpreting a given term can lead to serious consequences for applying scientific knowledge (Aplet and Cole 2010).

For example, the meaning of what is now labeled “naturalness” or described as “natural conditions”, central to conservation ecology and related fields, has been debated for more than a century and encompasses multiple interpretations (Aplet and Cole 2010). Another term fundamental to recreation ecology, *impact*, although more widely recognized, carries nuances that are often difficult to interpret and classify, including the type of impact, its amount, frequency, degree, magnitude, severity, spatial extent, and other descriptors of an undesirable/deleterious change to a natural resource caused by recreation (Hammitt et al., 2015; Monz et al., 2013). Moreover, terms that are directly applied in management, such as *restoration* and *rehabilitation*, continue to be debated with respect to their philosophical underpinnings and their practical application to the management of natural areas and associated infrastructure (Aplet and Cole, 2010; Hammitt et al., 2015; Gann et al., 2019).

Accordingly, across the various fields that intersect with recreation ecology, the meanings of key terms remain subject to active debate. Recent efforts have documented how some of these terms are used in the scientific literature (Aplet and Cole 2010; Hammitt et al. 2015). Within trail science, a systematic review of studies on trail degradation in protected natural areas (PNAs) and of strategies to reverse such degradation found substantial terminological variation (Simão et al. 2025). Some terms are applied consistently across studies, whereas others vary markedly in definition and scope. This linguistic diversity reflects the field’s interdisciplinary foundations and its intersections with adjacent domains, yet it creates conceptual and operational challenges. The use of ambiguous or overlapping terminology can hinder effective communication among stakeholders (Hsieh and Shannon 2005; Aplet and Cole 2010), thereby undermining the scientific foundation for sustainable trail management. Accordingly, the appropriate use of key terms enhances the comparability of studies, supports more robust literature reviews and evidence synthesis, and fosters better dialogue among all stakeholders involved in trail planning and management, including researchers, field technicians, land managers, funders, and lawmakers. It also helps to ground the drafting of legislation, policy documents, and funding proposals (e.g., Gerwing et al. 2022).

Drawing on foundations in trail science, recreation ecology and restoration ecology, we aim to clarify terminology relevant to trail science without prescribing rigid definitions. Specifically, we address four term sets: (i) terms for deleterious physical changes that affect trail usability (*impact, damage, degradation*); (ii) terms for the construction of new trails (*construction, build*); (iii) terms for the maintenance of existing trails (*maintenance, repair*); and (iv) terms for reversing trail degradation (*rehabilitation, restoration, renaturalization, recovery*), whether to return a trail to safe and functional use or to promote ecological restoration toward a reference ecosystem.

Based on this analysis, and in dialogue with influential literature across these domains (e.g., Aplet and Cole 2010; Monz et al. 2013; Gann et al. 2019; Marion 2023), we introduce two conceptual outputs: a model linking the *significance of deleterious physical change* to the *resilience* of trail ecosystems, and a terminology map that delineates areas of overlap between trail management and restoration ecology terminology. To translate these conceptual outputs into practice, we present a decision-making flowchart designed to foster consistent, conceptually grounded terminology and to support managers’ decisions about diverse trail management strategies in protected natural areas. By recognizing overlaps and transitions among the principal terms, managers are better positioned to identify ecological thresholds (Ma et al. 2013) and to determine when to prioritize interventions to maintain safe, functional trail use, or when to shift toward strategies that emphasize biodiversity conservation.

# Methodology

We conducted this study following the systematic literature review methodology proposed by Pickering and Byrne (2014), and in accordance with PRISMA reporting guidelines (Moher et al. 2009). Our sampling frame was the literature compiled by Simão et al. (2025), restricted to peer-reviewed scientific articles that examined at least one managerial, resource, social, or economic factor related to trail sustainability (Marion 2023), and that developed or evaluated actions or strategies to curb, mitigate, or reverse trail damage or degradation in protected natural areas (PNAs) worldwide.

Searches were performed in Google Scholar, Scopus, and Web of Science using the Boolean search string (trail OR trails) AND ("natural area*" OR "protected area*" OR "protected natural area*" OR park OR parks) AND ("restor*" OR "recover*" OR "rehabilit*" OR repair OR maintenance OR maintain* OR build*). The asterisk was used as a wildcard to capture suffix variations; for example, using “restor*” allows the search string to retrieve all possible endings such as “restoration,” “restored,” “restores,” and “restoring”. For Google Scholar, we screened abstracts in blocks of 50 and ceased screening once reviewers reached consensus that no relevant articles remained.

In total, we screened the abstracts of 1,020 articles and selected 28 original research articles and two literature reviews that met this study’s criteria (see Supplementary Information 1). Screening was performed independently by two reviewers; disagreements were resolved through discussion with a third reviewer. Duplicates were removed prior to screening. A PRISMA flow diagram summarizes the process (see Simão et al. 2025). The analyzed studies were conducted primarily in the United States (n = 11), but also in Australia (4), Japan (3), China (3), Poland (3), South Africa (1), Belgium (1), Canada (1), and Costa Rica (1), with most conducted in national parks (IUCN Category II - Dudley 2008) (see further details in Simão et al. 2025).

To analyze the terminology used in these 30 selected articles, we applied a summative qualitative content analysis (Hsieh and Shannon 2005) using a mixed deductive–inductive approach. Deductively, we enumerated occurrences of prespecified terms within four concept groups (*impact, degradation, damage; construction, build; maintenance, repair; rehabilitation, restoration, renaturalization, recovery*), recording (i) prevalence (number of articles in which a term appeared) and (ii) total occurrences. Only instances directly related to this study’s conceptual scope were included; for example, occurrences of impact that referred to statistical models were excluded. Inductively, we coded contextual meaning and mapped practices, whether or not explicitly labeled, to these concept groups and to reference frameworks in trail management and restoration ecology. This mixed-methods approach allowed us to explore not only the systematic quantification of terminology but also the identification of patterns in term usage and the clarification of the labels authors apply to practices and actions, yielding insights not readily attainable through literature review alone.

Additionally, we compared the contextual use of the terminology with definitions and principles found in key reference works in recreation ecology (Aplet and Cole 2010; Monz et al. 2013; Hammitt et al. 2015), restoration ecology (e.g. Gann et al., 2019; Gerwing et al., 2022) and trail science (e.g. Marion, 2023) to assess their consistency, nuances and alignment with practical and theoretical frameworks. It is important to note that the positive or negative value attributed to each term depends on the reference framework adopted by each author, for example, ecological conservation or trail functionality. We examined these terms within their original contexts, taking into account the value judgments conveyed by the authors. Our own assessments, grounded in the perspectives of trail science, recreation ecology and restoration ecology, are presented later in the Discussion and Management Implications sections. Finally, we developed a decision-making flowchart to operationalize a systematic approach for trail management in natural areas, integrating terminology and practices commonly used in the scientific literature.

# Results

## Quantitative analysis of term usage

*Impact*, *degradation*, and *damage*, the most prevalent terms across the dataset, were coded as descriptors of deleterious physical changes affecting trails. These terms appeared in more than 80% of the articles, and at least one of them was present in every article (Supplementary Information 1). Notably, impact stood out by accounting for over 650 occurrences (Fig. 1).

Terms related to the establishment of new trails, such as construction and build, appeared in 60% of the articles. Construction occurred far more frequently (over 200 occurrences) than build (approximately 20 occurrences). In contrast, terms related to the care of existing trails, including *maintenance*, *maintain*, and *repair*, were coded in nearly 90% of the articles, appearing at least once per article. Maintenance was particularly prominent, appearing more than 400 times throughout the dataset. Terms related to the reversal of trail degradation, including *recovery*, *restoration*, *rehabilitation*, and *renaturalization*, were coded less frequently overall. Recovery and restoration appeared in nearly half of the articles each with close to 200 occurrences, whereas rehabilitation and renaturalization were noted in less than 20% of the articles, each with less than 50 occurrences.


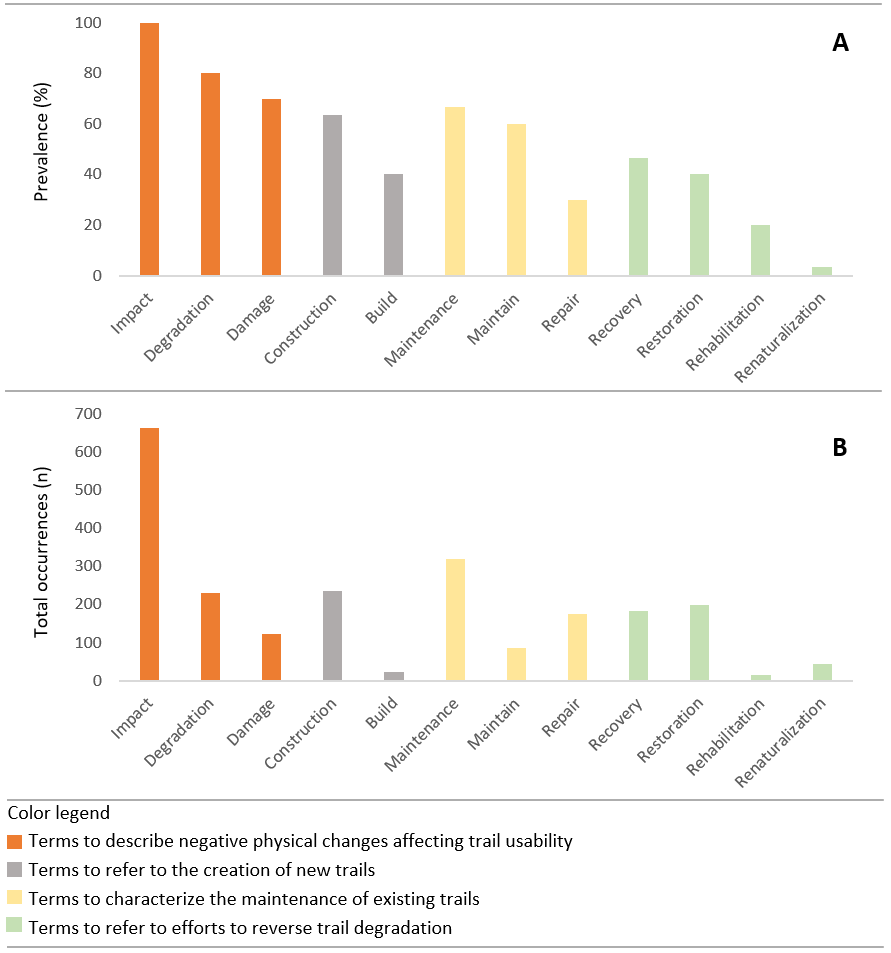


**Fig. 1** Term usage across the 30 articles analyzed. **A** Percentage of articles in which each term appeared. **B** Total number of occurrences of each term across all articles.

## Terms Used to Describe Physical Changes in Trails

Across the reviewed literature, the terms damage, impact, and degradation were coded in overlapping contexts to describe changes affecting various physical domains, particularly soil, trail tread, surrounding vegetation, water resources, and the broader environment. Less frequent codes linked these terms to cultural and historical features and to social aspects such as visitor experience. Co-occurrence patterns indicated recurring pairings between the three terms and specific domains (e.g., soil, vegetation, environment, trail environment) as well as with intensity/severity alteration qualifiers (low, medium, high, severe, variable). Across studies, explicit criteria for selecting one term over another were not reported. References to reversibility were heterogeneous: some changes were coded as temporary and likely reversible, whereas others were coded as long-lasting or irreversible (Table 1). The literature did not articulate criteria distinguishing which term would be more appropriate for reversible versus irreversible changes.

Impact, the most frequently used term across the dataset, was used to describe negative changes in multiple domains, particularly soil, the trail environment, and the broader natural environment. A passage from Marion (2023) exemplifies this predominantly negative application: “Severe trail impacts negatively affect the transportation and recreational utility of trails...” In this excerpt, the use of impact was coded as indicating negative effects across multiple domains, including trail functionality, resource protection, aesthetics, and visitor experience. It was also coded as reflecting varying severity, as seen in the expression “severe impacts”. Additionally, Olive and Marion (2009) provided an instance where impact was coded regarding significance and reversibility (irreversible) in the soil domain: "the loss of soil through erosion is generally considered a significant and irreversible form of trail impact." Considering effect, domain, intensity, and reversibility, the term damage was coded similarly to impact. In an excerpt from Tomczyk (2016), “… steps made of native rock and wood were installed […] minimizing damage to the trailside vegetation”, we coded damage to the vegetation domain and as indicating negative effect (trampling), with reduced intensity.

The term degradation was used across the literature with a meaning similar to impact and damage, typically referring to deleterious changes in various physical domains such as the trail tread and the soil. This usage is illustrated in Leung and Marion (1999), who state: “Major forms of trail degradation include wet muddy treads, tread widening…”. The excerpt exemplifies the association of degradation with trail and soil features. Similarly, degradation was coded in association with recreational pressure in Randall and Newsome (2008): “increasing recreational pressure from walkers has meant that walk trails worldwide have suffered accelerated degradation problems that diminish the natural, social and cultural values of an area …”. The term degradation was also coded for severity and potential reversibility. Ng et al. (2017), for instance, refer to “land degradation effects initiated by trail running events,” and further notes that “some types of trail degradation can recover within a few months”. In these excerpts we coded degradation with respect to both the severity of the changes and their potential for recovery over time. This relationship becomes even more explicit in Drewnik et al. (2019), who write: “… to determine the degree of soil degradation caused by hikers along tourist trails,” and later refers to management actions undertaken “to protect and restore degraded areas”. In these examples degradation was also coded with respect to severity and reversibility.

## Terms Used to Describe The Establishment of New Trails

Terms related to the deliberate establishment of new trails, namely construction and building, were coded as descriptors of interventions involving the planned creation of trail infrastructure. These interventions were typically coded as one-time projects implemented over a short timeframe as a single, planned actions. This use of construction was coded in Cahill et al. (2008), who write: “Constructing a well-designed trail or recreation site, surfacing it…,” and in Mende and Newsome (2006), who define constructed features as “an object constructed out of natural or artificial materials to maintain and manage the footpath”. These examples were coded as illustrating the association of construction with planned, purposeful trail establishment and infrastructure development. Similarly, occurrences of building were coded in contexts that reinforced this pattern. For instance, Schneller et al. (2020) referred to efforts “to build a new trail [...] to limit erosion”, and Tomczyk et al. (2016) mentioned the “building of wooden steps or logs”. In these cases, building was coding as describing targeted physical interventions aimed at establishing or reinforcing the trail system, often as a strategy to address environmental concerns such as erosion.

## Terms Used to Describe the Maintenance of Existing Trails

The terms maintenance and maintain were coded as descriptors of interventions aimed at preserving trail functionality (Table 2). This coding is evident in Mende and Newsome (2006), who state: “Without ongoing regular maintenance and upkeep of constructed features, trail conditions in the park will continue to deteriorate”. In addition, maintenance was also coded as referring to structural elements designed to protect trails, as seen in Leung and Marion (1999): “The effectiveness of maintenance features installed to divert water from trail treads”, and in Randall and Newsome (2008): “The installation of maintenance features such as boardwalks, water bars, and steps on sloped sections is crucial to sustainable trail management”. These examples were coded as illustrating the dual role of the term as both a management practice and a label for structural elements intended to preserve trail integrity. The excerpt from Kobayashi and Watanabe (2023) was coded as further illustrating maintenance as a preventive, regularly scheduled intervention focused on sustaining trail functionality: “Therefore, regular maintenance is necessary to ensure trail sustainability”.

The term repair, by contrast, was coded as a descriptor of corrective actions implemented occasionally in response to significant trail changes. This pattern was evident in Tomczyk et al. (2016): “Trails damaged by surface water runoff from the heavy rainfall, but which were subsequently repaired”, and in Ng et al. (2017): “…these trails will not be repaired unless obvious degradation is identified”. However, as observed with other terms, maintenance and repair were also coded as being used interchangeably. For example, Kobayashi and Watanabe (2023) note: “Trail maintenance through a combination of monitoring and repair work is vital…”.

**Table 1** Use of the terms impact, damage, and degradation across the analyzed articles (N = 30), including associated effects, domains, severity, and reversibility, and a summary definition based on content analysis.

| **Term^1^** | **Effect^2^** | **Domains^3^** | **Severity^4^** | **Reversibility^5^** | **Summary** |
| --- | --- | --- | --- | --- | --- |
| **Impact**  (30) | Negative (30) | soil (23), trail (21), vegetation (19), environment (15), social (quality of recreational experience, crowding, conflict, visitor safety) (6), aquatic environment (5), wildlife (4), historic resources (2), fauna (2), supply of ecosystem services (1), cultural resources (1), aesthetic values (1) | Variable levels (12) | Yes (16)  No (3) | Refers to any change that negatively affects the trail itself or its associated domains. This definition considers the possibility of varying degrees of intensity and potential reversibility. |
| **Damage** (21) | Negative (21) | vegetation (15), soil (11), trail (10), environment (3), ecological, physical, historical and cultural resources, trail features (logs, water bars) (1) | Variable levels (9) | Yes (16)  No (1) |  |
| **Degradation** (24) | Negative (24) | trail (18), natural resources (13), soil (13), vegetation (13), environment (6), cultural values/resources (2), recreational experience (2), water (2), historic resources, ecology, aesthetics (1), social values (1), wilderness (1), | Variable levels (11) | Yes (5)  No (1) |  |

Note: Numbers in parentheses indicate the number of articles (N = 30) in which the term was used in reference to: (^1^) changes in the trail environment; (^2^) negative or positive effects; (^3^) specific physical domains; (^4^) levels of severity (e.g., severely damaged, low impact, excessive impacts); and (^5^) reversibility or irreversibility of the alteration.

**Table 2** Use of the terms maintain, maintenance and repair across the analyzed articles (N = 30), including intervention type, focus, frequency, implementation timeframe, and a synthesized definition based on content analysis.

| **Term** | **Intervention Type** | **Focus** | **Frequency** | **Timeframe** | **Summary** |
| --- | --- | --- | --- | --- | --- |
| **Maintain or maintenance** | Preventive | Ensuring functionality over time | Regular, routine | Ongoing | The terms maintain/maintenance refer to continuous efforts to keep trails functional, safe, and in good condition over time, and are occasionally used as a synonym for repair. |
| **Repair** | Corrective | Fixing functionality | Occasional, when significant change occurs | Short-term or immediate | The term repair refers to actions taken to fix and stabilize existing trail changes to bring back trail functionality. It is occasionally used as a synonym for maintenance. |

## Terms Used to Describe the Reversal of Trail Degradation

The terms rehabilitation, restoration, recovery and renaturalization were coded as applied to the domains of vegetation, soil, and the trail tread/corridor and as spanning a continuum of human interventions from passive to active approaches. To clarify, we operationally defined passive approaches as those limited to restricting access or removing sources of disturbance (e.g., closures, fencing, regulatory prohibitions). In contrast, active or assisted approaches involve direct actions that modify environmental conditions to achieve the desired outcome, such as hydrological adjustments, terrain reshaping, or seeding and planting. This distinction aligns with the differentiation between natural (passive) and active (assisted) regeneration proposed by Gann et al. (2019).

In addition, these terms were coded in relation to varying timeframes and costs, distinct recreational objectives and diverse ecological focuses (Table 3). Recovery, the most frequently used term, was coded as primarily describing the spontaneous regrowth of vegetation without human intervention (passive) and as associated with the absence of intended recreational use thereafter. This coding is illustrated in Thurston and Reader (2001), who noted that “deciduous forest understory plants have high resilience (i.e., the ability to subsequently recover) when the recreational activity is not continuous”. Similarly, Roovers et al. (2005) refer to “vegetation recovery on footpaths in woodland that have been closed for access for 6 years” and Marion (2023) notes that “routing side-hill trails through grassy vegetation in sloping terrain is therefore more sustainable as trailside grasses will resist and more rapidly recover from occasional off-trail traffic”.

The terms restoration and rehabilitation were coded in the context of active interventions. Eagan et al. (2000), for example, describe the application of techniques aimed at the restoration of hydrology and soil conditions on degraded abandoned trails: “The project restored the natural hydrology and soils [...] of abandoned trail…”. In a similar vein, in Kobayashi and Watanabe (2023) rehabilitation was coded as describing the removal of eroded sediments deposited over surrounding vegetation: “The repair work transported a large amount of sediment to the upper section to rehabilitate the alpine vegetation covered by sediment”. However, restoration was also coded in passive contexts; in some cases, it referred to strategies without direct intervention. Tomczyk et al. (2016), for instance, write: “Leaving trails for natural restoration…”. The term renaturalization, found only in Drewnik et al. (2019), was coded specifically in the soil domain and exclusively in contexts of passive intervention, such as closing or fencing off parts of tourist trails. The authors aimed to “analyze the direction and the rate of renaturalization of degraded soils experiencing renaturalization for various periods of time”.

**Table 3** Summary of key terms used in the literature to describe trail recovery processes, including ecological domain, timeframe, cost, intended recreational use after the process, intervention focus and approach, and expected impact on biodiversity. Content derived from literature-based content analysis.

| **Term** | **Domain** | **Timeframe** | **Cost** | **Intended Recreational Use After Process** | **Intervention Focus** | **Intervention approach** | **Expected Ecological outcomes** |
| --- | --- | --- | --- | --- | --- | --- | --- |
| **Recovery** | Vegetation (mainly); trail, occasionally soil | Medium to long-term | Low | Typically restricted | Gradual reestablishment of vegetation or trail condition, often aiming for partial return to previous state, with limited intervention. | Mainly passive - trail closure, fencing, monitoring, limited use or natural regrowth. | Low to moderate biodiversity gains; long-term improvement depends on site conditions and prior degradation levels. |
| **Restoration** | Vegetation, trail, soil, hydrology, topography | Medium to long-term | Medium–High | Occasionally allowed | Reconstruction of ecological integrity, including native vegetation, soil structure, and hydrological functions. | Active (occasionally passive) - water bars, native planting, brushing, topographic reshaping, soil stabilization, fencing (passive) | High; potential full ecosystem reestablishment and enhancement of biodiversity. |
| **Rehabilitation** | Trail (mainly), vegetation, soil, ecosystem services | Short to medium-term | Medium–High | Occasionally allowed | Improvement of ecological functions or trail usability, without fully recovering the original ecosystem. | Active - drainage correction, erosion control, surface stabilization, sediment removal. | Moderate; supports ecosystem services and trail usability. |
| **Renaturalization** | Soil | Medium to long-term | Low | Occasionally allowed | Reactivation of natural soil processes with minimal human action. | Passive - trail closing, abandonment, fencing | Gradual improvement limited to soil and initial vegetation strata. |

# Discussion

## Trail terminology as an indicator of diverse research focuses in trail science

The analysis of term prevalence and total occurrence across the reviewed literature suggests that trail science has generated more evidence on understanding and monitoring deleterious environmental change and the factors related to these changes, as reflected by the high prevalence of terms such as *impact*, *damage*, and *degradation*. In contrast, the lower prevalence of terms associated with the care of trails (*maintenance*, *repair*), and with the reversal of deleterious changes (*restoration*, *rehabilitation*, etc) suggests that fewer studies have been published on these themes. This outcome was expected, given that the established scope of recreation ecology and trail science places the understanding of impacts at the core of these fields. Nevertheless, from a broader perspective, the goals of these fields also encompass pursuing more effective approaches to managing recreation areas and associated infrastructure, such as trails (Hammitt et al. 2015; Marion 2023). This pattern is consistent with the findings of Sumanapala and Wolf (2019), who reported in a review of recreation ecology that management tools designed to reduce impacts are less known, in part because most research has focused on short-term negative impacts at recreational sites. Accordingly, our analysis highlights the need for further long-term research and dissemination on maintenance practices that keep trails in good condition and on strategies to reverse visitation-related trail degradation, particularly within trail systems.

Addressing the need to keep trails in good condition and to reverse visitation-related degradation requires not only identifying the factors that drive trail degradation but also developing, testing, and refining effective techniques to sustain trail infrastructure and adjacent ecosystems in good condition over time (Ng 2022). It is equally important to establish strategies for managing degraded trails, whether by returning them to safe, functional use (e.g., Ramos-Scharrón et al. 2014; Tomczyk et al. 2016; Kobayashi and Watanabe 2023) or by facilitating ecological restoration in areas where recreational use should be discontinued to enable the natural ecosystem to be reestablished as fully as possible (e.g., Eagan et al., 2000; Ramos-Scharrón et al., 2014; Ng et al., 2017; Drewnik et al., 2019). Moreover, it is essential to recognize that trail management is embedded within broader multiscale social–ecological systems, and that recreation, while capable of generating negative ecological changes, may also contribute positively to conservation outcomes, as articulated in Miller et al. (2022) through the concept of recreation–ecosystem interactions.

## Conceptual Model of Trail-Related Ecosystem Changes

Across the reviewed literature, the terms impact, damage, and degradation were used interchangeably to describe deleterious changes affecting the broader trail ecosystem, including surrounding vegetation, soil, and the trail tread/corridor itself. We consider the meanings ascribed to these three terms to fit the definition of impact presented by Hammitt et al. (2015) within recreation ecology, a definition that entails a value judgment—i.e., deleterious physical change denotes an undesirable change from a particular standpoint. Because the terms were used as synonyms, no intrinsic gradient of significance among them could be identified. When authors wished to differentiate severity, degree, spatial extent, frequency, or persistence, they relied on qualifiers such as “high,” “low,” “large,” “severe,” “frequent,” and “reversible,” among others. It is important to note that, even when such qualifiers are applied, the assessment of significance still depends on the evaluator’s perspective. What is considered a high impact from a land manager’s standpoint could be viewed as low by recreationists (Hammitt et al. 2015).

However, communication across the scientific domains related to trail management could benefit from having terms that, on their own, convey a gradient of significance, even though condensing the multiple characteristics noted above (e.g., severity, degree, spatial extent, reversibility) into one, in this case, significance, or a few terms is inherently difficult. Here, the *significance of a deleterious change* can be understood and measured using the same variables that represent “increasing impact” or “increasing disturbance” in Monz et al. (2013), such as soil erosion and trampling of vegetation.

A comparable gradient already appears in ecological restoration. These same three terms also appear in the Second Edition of the Society for Ecological Restoration’s *International Standards* (SER Standards; Gann et al., 2019) to characterize deleterious ecosystem changes. They are, for example, embedded in the official definition of ecological restoration as “the process of assisting the recovery of an ecosystem that has been degraded, damaged, or destroyed”. In the SER glossary damage is defined as “an acute and obvious deleterious impact on an ecosystem”; degradation as “a level of deleterious human impact to ecosystems that results in the loss of biodiversity and simplification or disruption in their composition, structure, and functioning, and generally leads to a reduction in the flow of ecosystem services”; and destruction as the condition in which “degradation or damage removes all macroscopic life, and commonly ruins the physical environment of an ecosystem”. Taken together, these definitions imply an ascending gradient of significance: damage refers to acute and evident alteration; degradation denotes more extensive deleterious change with broader ecological consequences; and destruction represents the most extreme condition.

Acknowledging this gradient of significance can improve how negative alterations are described and compared across studies, although cross-study comparisons obviously require far more detailed qualitative and quantitative descriptions of the identified changes. Moreover, even within the definitions proposed by Gann et al. (2019), there is an area of overlap among the terms, which makes it counterproductive to attempt to establish rigid concepts and fixed boundaries for them. However, one can conceptually represent transitions in significance among these terms by grounding them in a factor that is ecologically and managerially relevant and closely related to many of the descriptors noted above. That factor is *resilience*, which refers to the capacity of an ecosystem to absorb disturbance and reorganize its core structure, function, and identity without external intervention (Begon et al. 2014; Gann et al. 2019; Llena et al. 2024). Accordingly, as changes in an ecosystem increase in magnitude, frequency, spatial extent, and degree, its capacity to absorb those alterations and to reorganize without external intervention declines. In other words, there is a negative relationship between the significance of the alteration and resilience: the greater the significance of the change, the lower the ecosystem’s resilience.

Viewed through this lens, the assessment becomes less dependent on the evaluator’s value judgment, because it relies not on an individual’s perception of an impact (see Hammitt et al. 2015) but on the ecosystem’s capacity to absorb disturbances and reorganize. Additionally, considering the significance of change, rather than proxies such as amount of use, reduces confounding arising from different use–impact response curves along increasing-use gradients (see Monz et al. 2013). Thus, relating the significance of environmental change to ecosystem resilience makes the ecosystem itself the common reference for both variables.

In addition, by adding to this conceptual analysis a distinction between reversible and irreversible alterations, it becomes possible to identify critical points where the need for management intervention is imminent. To support this distinction, we refer to the concept of an *ecological threshold*, a concept commonly used in ecology (e.g., Ma et al. 2013) and also adopted by restoration ecology. An ecological threshold represents a tipping point beyond which further increases in impact cause an ecosystem to lose its ability to regain its previous state or trajectory without substantial human intervention. If no such intervention is made, degradation may continue until it becomes effectively irreversible, ultimately leading the system to shift toward an alternative stable state.

Translated to the context of trail science, this framework can be described by the following scenario: under low-significance alterations, the tread surface may initially operate in a quasi–steady-state volumetric sediment budget in which the small volume of material removed by the use and diffuse runoff is roughly offset by lateral inputs of loose particles and organic detritus (i.e., the ecosystem’s capacity to absorb change and reorganize without external intervention). However, poorly planned trails with high-significance alterations can push the system past the ecological threshold. Once this point is crossed, rainfall runoff becomes increasingly channeled along the trail, exacerbating erosion beyond the rate of sediment replenishment. In such cases, erosion tends to accelerate unless corrective human interventions, such as reshaping the microtopography of the trail tread, are implemented to interrupt the positive feedback loop of concentrated runoff.

To illustrate this scenario, we propose a conceptual model to support the use of terminology to describe alterations by distinguishing the significance of deleterious physical change according to system resilience. In the model, *resilience* is represented on the vertical axis and decreases as the *significance of deleterious change* increases along the horizontal axis (Fig. 2). We follow the gradient identified in Gann et al. (2019), adding the term impact as representing the lowest-significance alterations. Thus, systems that are impacted or damaged exhibit low to moderate alteration while maintaining high resilience. As alteration intensifies, the system enters a degraded state, where structure or function is compromised and resilience is reduced, requiring increasingly active intervention to regain structure and function. Eventually, an ecological threshold is crossed, beyond which degradation becomes irreversible, and the system enters a destroyed state, having lost the capacity to return to a previous state without substantial human effort (see Ma et al., 2013). This conceptual model illustrates how these terms correspond to different stages along a continuum of deleterious changes and supports more consistent and meaningful terminology use in trail management literature.


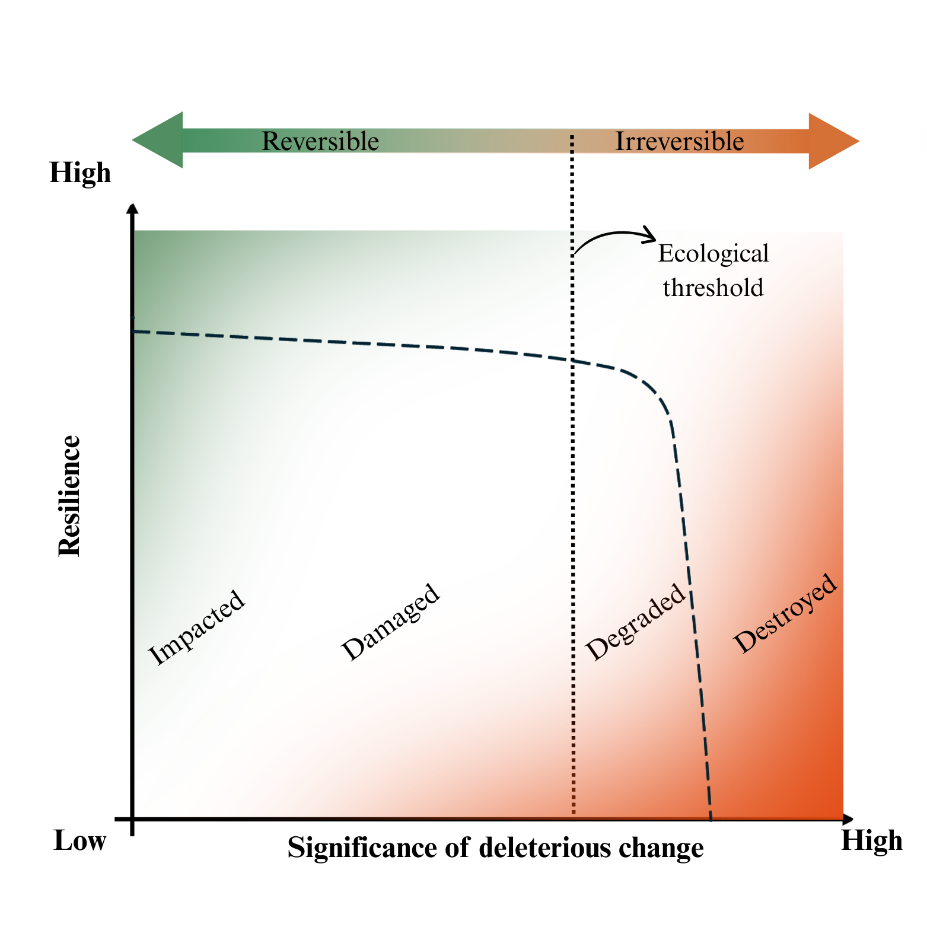


**Fig. 2** Conceptual model linking the significance of deleterious physical change to trail ecosystem resilience. Minor and localized changes (impact) and moderate changes (damage) fall within a reversible region, as the ecosystem retains the capacity to regain its characteristics without human intervention. Once the significance of deleterious physical change increases and an ecological threshold is crossed, the ecosystem’s condition becomes effectively irreversible, and the system shifts into degraded or even destroyed states. Gradient colors, rather than discrete categories, were intentionally used to indicate that transitions between one term and another, and between reversible and irreversible, do not have fixed boundaries but instead form a transition zone.

Based on this conceptual model, a trail exhibiting signs of minor significance of alteration (e.g., limited surface erosion or occasional water pooling) could be considered impacted or damaged (e.g., Hockett et al. 2017). If such changes become more significant, for example when surface erosion develops into rill formation with complete channeling of rainwater, or when entire sections of the trail remain flooded for extended periods, the trail may be classified as degraded and would require immediate repair measures (e.g., Ramos-Scharrón et al. 2014). In more extreme cases, such as a landslide that displaces the entire trail tread downslope or when segments become persistently inundated and lose their functionality altogether (e.g., Tomczyk et al. 2016; Kobayashi and Watanabe 2023), these sections may be classified as destroyed, having lost their functionality, ecological integrity, and the capacity to regain their characteristics (i.e., resilience) without human intervention.

## Terms Used to Describe the Establishment of New Trails and the Maintenance of Existing Trails

The terms building and construction were coded as used interchangeably to denote the planned creation of new trails or associated features. This pattern is also evident in the technical literature on trail management. For example, on the websites of American Trails, a coalition of trail-related organizations, agencies, and individuals, and of the International Mountain Bicycling Association (IMBA), these terms are consistently presented as synonyms (American Trails 2007; IMBA 2025). This suggests a high degree of terminological standardization across both scientific and technical sources.

The terms maintenance and repair, although sometimes coded as being used interchangeably, show a clear pattern in their association with the type and timing of intervention. The term maintenance typically refers to preventive actions performed regularly to preserve trail functionality, while repair is more commonly associated with corrective actions applied occasionally in response to significant damage. For example, Marion (2023) notes the importance of designing trails to be “permanently self-draining with the goal of avoiding the need for annual maintenance”, reflecting a routine preventive approach. In contrast, Kobayashi and Watanabe (2023) describe repair techniques used to stabilize eroded sections of trails using soil-filled palm-fiber bags and wooden steps, an example of a short-term corrective intervention. Based on these usage patterns, maintenance is generally linked to less significant changes (e.g., impacted or damaged trails), whereas repair tends to be applied in contexts involving more advanced changes, such as degraded or destroyed trail segments. Because these terms were most often used within similar patterns, we propose maintaining this usage in trail science. Accordingly, maintenance should continue to denote routine care of trails, encompassing corrections of low-significance deleterious changes, whereas repair should continue to denote more targeted, problem-specific interventions to correct higher-significance deleterious changes.

## Terminology for Reversing Trail Degradation: Insights from Restoration Ecology

Among the term sets analyzed in this article, those related to the reversal of physical trail alteration (rehabilitation, restoration, recovery, and renaturalization) warrant particular attention. The appropriate use of these terms depends fundamentally on the management goals established for a given area or trail (see The Dilemma of wilderness Management in Aplet and Cole 2010). Setting these goals requires consideration of multiple factors, including stakeholder aspirations, available financial and technical resources, and applicable regulatory frameworks (Hobbs and Harris, 2001). In the context of trail management, the first step is to determine whether the reversal of alterations intended to enable continued, safe trail use or to decommission the trail, that is, an explicit management decision.

The SER International Standards (Gann et al. 2019), although primarily focused at broader ecosystem landscape scales, offer useful definitions to select precise terminology for the reversal of trail alteration. While these definitions were developed within restoration ecology, incorporating them can enhance clarity and consistency in trail science terminology, given the overlap between these fields. According to the SER Standards, there is a continuum of activities and interventions, referred to as the *restorative continuum*, along which environmental conditions are progressively improved through different types of actions, from rehabilitation to restoration, ultimately aiming for full recovery.

For example, r*ehabilitation* is defined as “management actions that aim to reinstate a level of ecosystem functioning on degraded sites, where the goal is renewed and ongoing provision of ecosystem services rather than the biodiversity and integrity of a designated native reference ecosystem” (Gann et al., 2019). Thus, when the goal is to reestablish safe, functional trail use, rehabilitation is the most appropriate term within this framework. In such cases, the primary objective is to sustain key ecosystem processes and the continued delivery of ecosystem services. In the context of trails, this includes the provision of cultural ecosystem services associated with recreational trail use (MEA 2005; Tomczyk et al. 2016).

However, when the management goal is to permanently discontinue trail use and guide the area toward the biodiversity and integrity of a designated native ecosystem, the concept of trail rehabilitation, focused solely on reestablishing functionality, becomes insufficient. In such cases, the definitions of ecological restoration and recovery, become especially relevant. To interpret these definitions properly, it is first necessary to introduce the concept of a *reference ecosystem*: “a representation of a native ecosystem that is the target of ecological restoration... A reference ecosystem usually represents a nondegraded version of the ecosystem…” (Gann et al. 2019).

With this concept in mind, it is important to emphasize that the target of ecological restoration is not to pursue a “natural,” “original,” or “pristine” condition (naturalness) for the site, since directing a system toward such a state is highly problematic (Aplet and Cole, 2010). Areas we perceive as “natural” or “pristine” have often been managed, more or less intensively, by humans, and what appears natural may in fact reflect centuries or millennia of human–environment interactions (e.g., Levis et al., 2017). One might propose defining the representative nondegraded state as the condition that existed prior to trail establishment; however, determining that state is challenging because many trails in use today are centuries old (Erickson, 2009; Snead et al., 2009; Gou and Shibata, 2017), so the pre-trail condition is often unknown. Moreover, steering a site toward a “pristine” state could require continuous active human intervention, entailing disproportionate effort and resources, and in light of ongoing global climate change this is even less plausible (Aplet and Cole, 2010). Accordingly, for trail management we propose that the surrounding ecosystem with minimal observable alterations can serve as the *reference ecosystem*.

Building on these foundational concepts, we now present the remaining definitions from the SER International Standards. Ecological restoration is defined as “the process of assisting the recovery of an ecosystem that has been degraded, damaged, or destroyed,” while recovery is “the process by which an ecosystem regains its composition, structure, and function relative to the levels identified for the reference ecosystem. In restoration, recovery usually is assisted by restoration activities; and recovery can be described as partial or full” (Gann et al., 2019). These definitions underscore that ecological restoration entails deliberate (active) human interventions intended to facilitate or accelerate recovery, with the goal of achieving partial or full recovery of the ecosystem. Accordingly, when the management goal is to permanently discontinue trail use and promote biodiversity conservation, the term restoration should be applied to techniques undertaken to facilitate partial or full recovery of the site. This usage is consistent with the conceptualization of restoration discussed by Aplet and Cole (2010). However, unlike portrayals that equate restoration with a return to a “pristine” state, here restoration is explicitly oriented toward a reference ecosystem, which may, and likely will, differ from any presumed pristine ecosystem.

On the other hand, recovery is interpreted as a passive outcome, or as a state along a continuum, either partial or full, resulting from that process. Notably, this continuum corresponds to what Aplet and Cole (2010) termed drift and recovery, respectively. In their conceptual model, a site under passive management tends toward a more novel condition (drift), whereas under active management it can be directed toward conditions closer to the presumed pristine condition (recovery). Because we treat recovery as an outcome or state in this study, it does not appear as a distinct intervention in our conceptual tools; rather, it is represented as an outcome/state, not an action.

To further clarify these relationships, we propose a terminology map (Fig. 3) that illustrates how these terms fit within the contexts of trail management and restoration ecology. The terms repair, rehabilitation, and restoration overlap substantially with elements of restoration ecology, especially when management practices focus on conserving biodiversity and ecological processes through a variety of interventions and on supporting ecosystem recovery.

Examples of interventions commonly used in restoration ecology that can also be applied to the ecological restoration of degraded trails where recreational use is no longer allowed include both passive strategies (e.g. area closure or abandonment to allow natural regeneration) and active (assisted) or so-called artificial techniques (Ma et al. 2013), such as invasive-species control, seeding and planting of native grasses, shrubs, and trees, translocation of soil and litter seed banks, installation of artificial perches and reintroduction of native fauna, all commonly associated with restoration ecology (Martins 2012; Nelson et al. 2024).


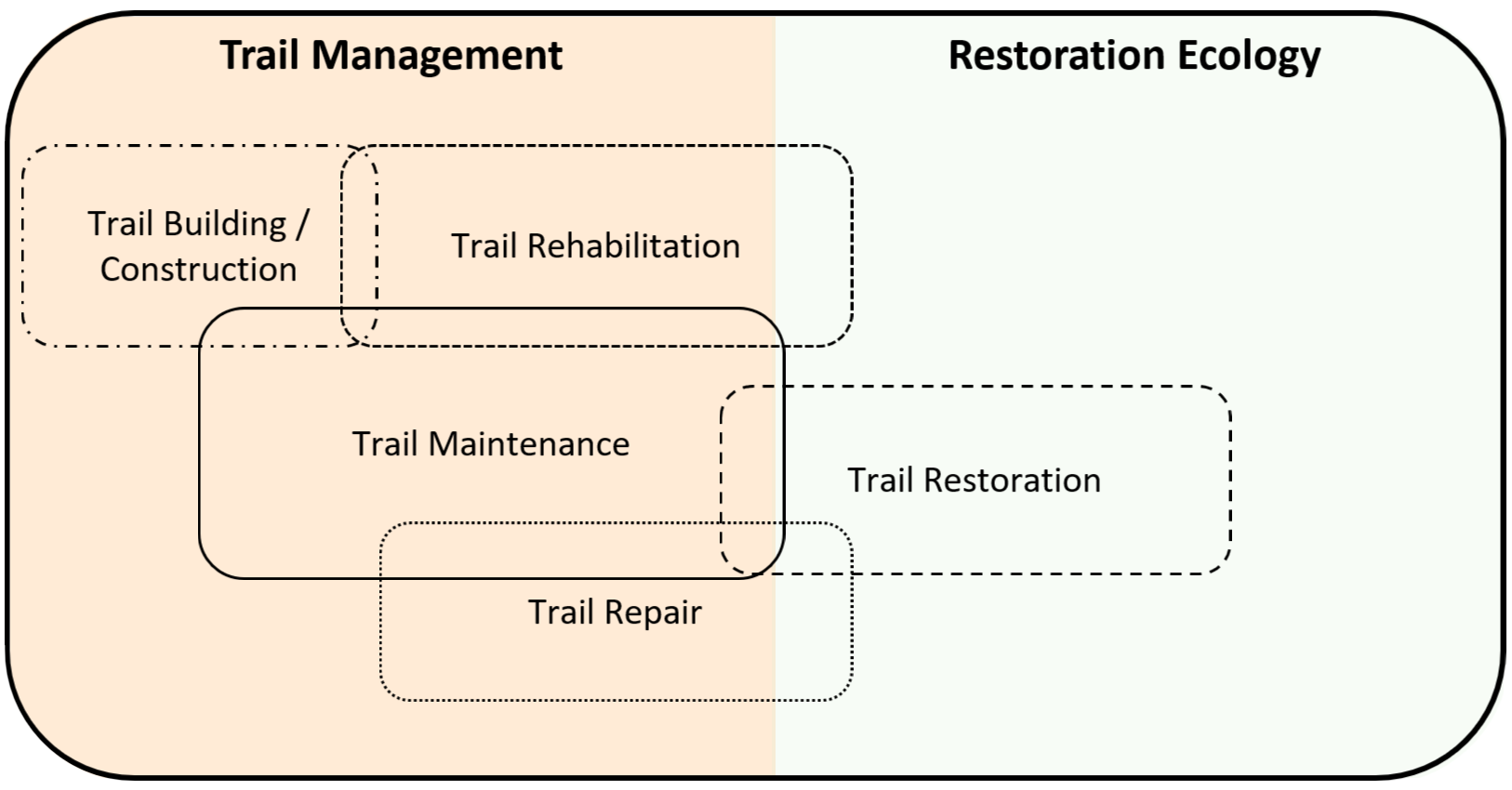


**Fig. 3** Terminology map across trail management and restoration ecology; overlaps indicate that the same activities are referenced by multiple terms and may be applied in both domains.

## Management Implications: A Decision-Making Framework for PNA Managers

Trail sustainability requires a holistic approach that considers multiple factors, such as managerial, ecological, social and economic elements (Campbell 2024; Marion 2023). Managers of PNAs must address conflicts both within and among these elements when making decisions about trail management. It is their responsibility, ideally in collaboration with advisory or decision-making boards, where such bodies exist, and after considering the needs and aspirations of society, to decide how to manage existing trails and whether to establish new ones (Schneller et al. 2020). Decision-making inevitably entails actions, or deliberate non-actions, that align with the terminology discussed in this article.

At times, given the many possible courses of action, it becomes difficult to evaluate all scenarios and arrive at a defensible decision. Moreover, once a decision is made, communicating it to stakeholders responsible for subsequent activities (e.g., trail maintenance, rehabilitation, restoration) can be challenging and may lead to misunderstandings. Accordingly, a targeted, stepwise analysis that relies on clearly defined terminology and is supported by a visual decision-support resource, such as a decision-making flowchart, can be highly effective. Such a tool helps structure the analysis, increases confidence in decisions, and reduces the risk of misunderstandings among stakeholders (e.g., PNA managers, state regulatory agencies, contractors, and nonprofit organizations), thereby improving communication.

At this stage of the article, having clarified the definitions, understanding and relationships of the key terms commonly used in trail management, and how these terms draw from recreation ecology, trail science and restoration ecology, we present a flowchart to support decision-making in outdoor recreation areas, where the establishment, management or deactivation (closure) of trails may be under consideration (Fig. 4).

The decision-making flowchart begins with the assessment of the management unit to assess whether any trails are present. If no trails exist, managers must evaluate the need to establish a new one. If a trail is deemed unnecessary, the area should be kept trail-free to avoid further resource alteration. If the creation of a trail is justified, the next step involves trail planning and construction, with due consideration of environmental suitability and management objectives (see Leung and Marion 1999; Marion 2016).

For areas with existing trails, the flowchart requires managers to first decide whether the trail is still necessary. If the trail is no longer needed, the next step is to deactivate the trail and then assess its condition. Trails with a low level of impact can be closed and left to passive restoration, allowing for potential partial or full ecosystem recovery (e.g., Eagan et al. 2000; Tomczyk et al. 2016). If the trail is impacted or damaged, indicating moderate disturbance remains, active (assisted) restoration interventions may be required to facilitate recovery, such as replanting native vegetation or stabilizing soils (e.g. Nelson et al. 2024). In cases where the level of trail change has crossed the ecological threshold, i.e., the trail has reached a degraded or destroyed state, more intensive restoration measures are necessary to promote soil stabilization and ecological recovery (see Bratton et al. 1979).

If a trail is still needed in the area, managers should assess the current condition of the trail. For impacted or damaged trails (showing moderate-significance alterations), ongoing maintenance may be sufficient to preserve functionality and prevent further degradation (see IMBA 2004; IMBA 2007; Marion 2023, which provide practical guidance on trail maintenance). In the case of degraded or destroyed trails, managers may opt for rehabilitation, if the trail needs to be in the same place, particularly where it has historical or cultural value (e.g. Gou and Shibata, 2017). In other cases, they may decide to redesign the trail. Redesigning often involves constructing a new segment and deactivating the most impacted section, which then requires active (assisted) restoration interventions to halt further degradation and facilitate ecosystem recovery.


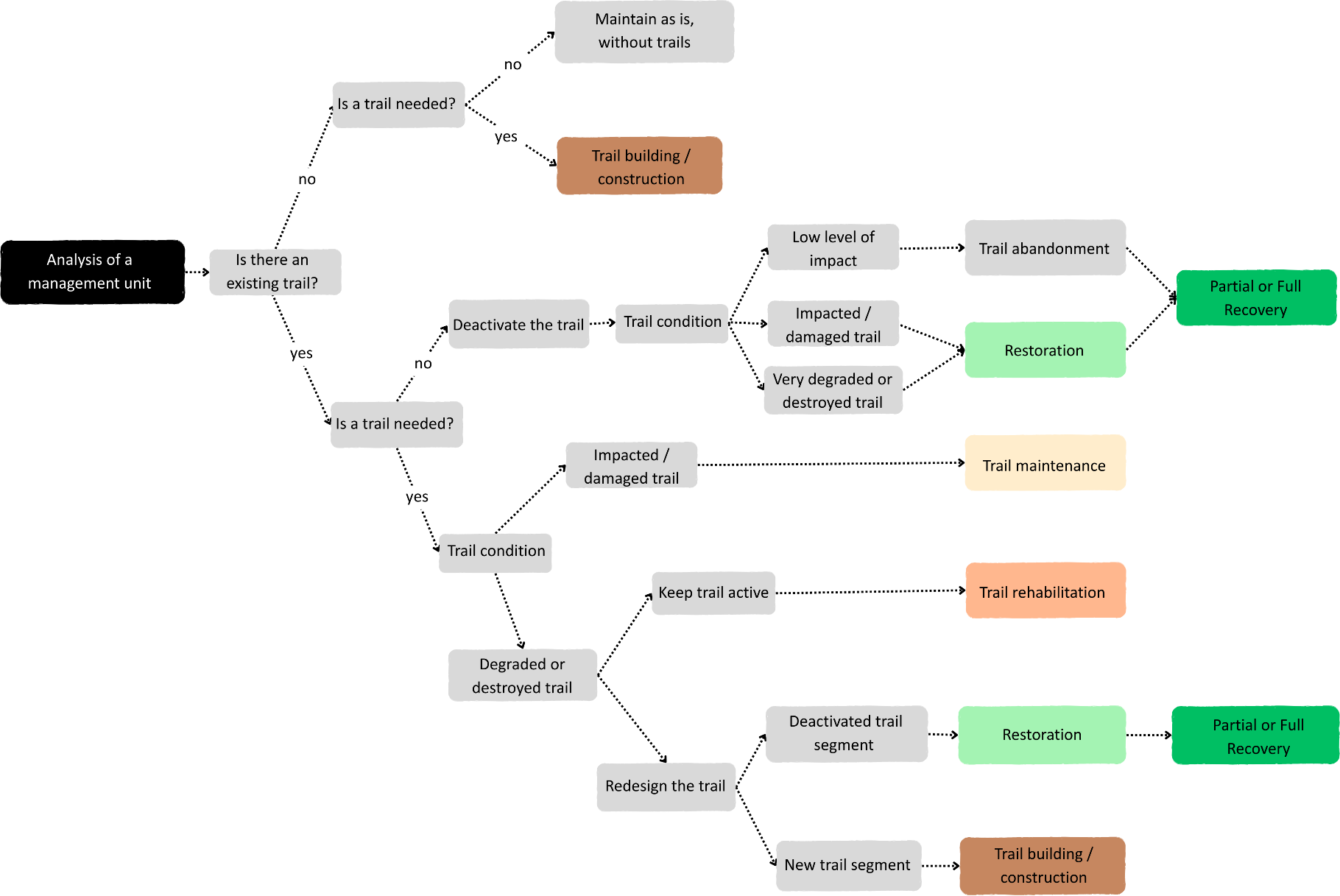


**Fig. 4** Decision-making flowchart for the management of recreational trails in protected natural areas, illustrating decision pathways and recommended actions according to trail presence/necessity, trail condition (“impacted,” “damaged,” “degraded,” “destroyed”) and management objectives (continued use vs. deactivation/closure). Each color represents an intervention type (e.g., construction, maintenance, repair, rehabilitation, restoration) or an outcome (e.g., trail kept closed or trail-free).

We believe this flowchart can help managers gain a broader, clearer perspective on the range of options for managing recreational use in specific zones of PNAs under their jurisdiction. Furthermore, a solid grasp of terminology can improve the ability to identify both effective and ineffective management practices in the scientific literature, thereby strengthening decision-making for these areas. This knowledge can also assist policymakers, lawmakers, practitioners and researchers involved in the governance and sustainable use of protected natural areas.

# Conclusion

This study documents substantial variability in how trail-related terminology is used, especially in reference to deleterious change and its reversal. Although *impact*, *damage*, and *degradation* are widely employed to describe deleterious physical changes that affect trail usability, their use, by itself, rarely indicates the level of significance of deleterious change. Likewise, terms associated with reversing degradation (*rehabilitation*, *restoration*, *renaturalization*, *recovery*) are often applied without consistent criteria, which obscures management goals and expected outcomes.

By synthesizing evidence from trail science and analyzing the terminology in light of restoration ecology and recreation ecology we provide a clearer basis for consistent communication. The conceptual model introduced here positions key terms along a gradient that links the *significance of deleterious change* to *resilience*. The terminology map shows how terms from trail science and restoration ecology relate to one another and where they overlap. Finally, the decision-making flowchart translates this framework into practice, guiding assessment and the selection of interventions according to explicit management goals.

Improving terminological clarity is essential for advancing scientific understanding and for enabling coherent communication among researchers, land managers, policymakers, and other stakeholders. In recreation ecology and trail management, using clear, context-specific terminology aligned with management pathways and goals can help identify *ecological thresholds*, support timely decisions, and promote more sustainable trail management outcomes.

**Limitations**

One limitation of this study is that, as discussed by Aplet and Cole (2010), the meaning and use of technical terms are continually evolving; therefore, although clarifying their definitions is important, imposing fixed, rigid definitions may be counterproductive. Another important limitation concerns one of this article’s key concepts: the significance of deleterious change. Variables related to impacts in recreation ecology, such as amount of use (see Monz et al., 2013), type of use, and spatial extent, are relatively straightforward to measure (e.g., number of people passing along a trail; hikers versus motorized vehicles; linear meters affected, respectively). By contrast, resilience, the term used here to relate the significance of deleterious alteration, is more complex to quantify and may require long-term experimental study. Even so, in the management of protected natural areas, trail management, and in restoration ecology, a central question often concerns an ecosystem’s capacity to regain its characteristics without human intervention or, alternatively, the level of human effort required to manage that system. Thus, reflecting, even at a conceptual level, on how the significance of an impact relates to an ecosystem’s resilience (the trail and its surroundings) can broaden the range of management options for these areas. Finally, it is important to note that the conceptual model and the decision-making flowchart are theoretical simplifications of reality. Consequently, external factors can influence the assessment of a trail’s condition (its significance of deleterious change and its resilience) and decision-making about trail management. Accordingly, a landscape-ecology analysis of the setting in which the trails are embedded should be incorporated into assessments.

**Data Availability**

Data are provided within the manuscript or supplementary information file.

# Acknowledgments

We thank the Federal Institute of Education, Science and Technology of Amazonas (IFAM), Tefé Campus (Brazil), for granting the first author, a federal public servant and faculty member, academic leave for training pursuant to Law No. 8,112/1990. Manel Llena is affiliated with the Fluvial Dynamics Research Group (RIUS), a Consolidated Group recognized by the Generalitat de Catalunya (2021 SGR 01114). We also acknowledge support from the CERCA Program of the Generalitat de Catalunya (Spain). We are especially grateful to the reviewers for their fundamental contribution to improving this manuscript; their recommended literature from recreation ecology, critical comments, questions, and suggestions substantially enhanced the clarity and organization of the current version.

**Author Contributions**

All authors contributed to the conception and design of the study. Material preparation, data collection, and analysis were performed by M.V. and E.I. The first draft of the manuscript was written by M. V., and E.I. and M.L. provided substantial input and critical revisions to previous versions. All authors read and approved the final manuscript.

**Conflict of Interest**

The authors declare no competing interests.

# References

American Trails Staff (2007) Basic elements of trail design and trail layout. [https://www.americantrails.org/resources/basic-elements-of-trail-design-and-trail-layout. Accessed 10 Aug 2025](https://www.americantrails.org/resources/basic-elements-of-trail-design-and-trail-layout.%20Accessed%2010%20Aug%202025).

Aplet GH, Cole DN (2010) The trouble with naturalness: Rethinking park and wilderness goals. In: Cole DN, Yung L (eds) *Beyond naturalness: Rethinking park and wilderness stewardship in an era of rapid change*. Island Press, Washington, DC, pp 12–29.

Begon M, Howarth RW, Townsend CR (2014) *Essentials of Ecology*, 4th edn. Wiley-Blackwell, Chichester, UK.

Bratton SP, Hickler MG, Graves JH (1979) Trail erosion patterns in Great Smoky Mountains National Park. *Environmental Management* 3(5):431–445. https://doi.org/10.1007/BF01866582

Cahill KL, Marion JL, Lawson SR (2008) Exploring visitor acceptability for hardening trails to sustain visitation and minimise impacts. *Journal of Sustainable Tourism* 16(2):232–245. <https://doi.org/10.2167/jost804.0>

Campbell T, Kirkwood L, McLean G, Torsius M, Florida-James G (2021) Trail use, motivations, and environmental attitudes of 3780 European mountain bikers: what is sustainable? *International Journal of Environmental Research and Public Health* 18(24):12971. <https://doi.org/10.3390/ijerph182412971>

Coleman RA (1977) Simple techniques for monitoring footpath erosion in mountain areas of north west England. *Environmental Conservation* 4(2): 145-148. https://doi.org/10.1017/S0376892900025443

Drewnik M, Musielok Ł, Prędki R, Stolarczyk M, Szymański W (2019) Degradation and renaturalization of soils affected by tourist activity in the Bieszczady Mountains (South East Poland). *Land Degradation & Development* 30:670–682. <https://doi.org/10.1002/ldr.3231>

Dudley N (ed.) (2008) Guidelines for Applying Protected Area Management Categories. Gland, Switzerland: IUCN. x + 86 pp. ISBN 978-2-8317-1086-0.

Eagan S, Newman P, Fritzke S, Johnson L (2000) Restoration of multiple-rut trails in the Tuolumne Meadows of Yosemite National Park. In: Cole DN, McCool SF, Borrie WT, O’Loughlin J (eds) *Wilderness Science in a Time of Change Conference — Volume 5: Wilderness Ecosystem, Threats, and Management*. USDA Forest Service Proceedings RMRS-P-15-VOL-5, Rocky Mountain Research Station, Ogden, UT, pp *188–*192.

Erickson CL (2009) Agency, Causeways, Canals, and the Landscapes of Everyday Life in the Bolivian Amazon. In: Snead JE, Erickson CL, Darling JA (eds) *Landscapes of Movement: Trails, Paths, and Roads in Anthropological Perspective*. University of Pennsylvania Museum of Archaeology and Anthropology, Philadelphia, pp 204–231.

Gann GD, McDonald T, Walder B, Aronson J, Nelson CR, Jonson J, Hallett JG, Eisenberg C, Guariguata MR, Liu J, Hua F, Echeverría C, Gonzales E, Shaw N, Decleer K, Dixon KW (2019) International principles and standards for the practice of ecological restoration: second edition. *Restoration Ecology* 27(S1):S3–S46. https://doi.org/10.1111/rec.13035

Gerwing TG, Hawkes VC, Gann GD, Murphy SD (2022) Restoration, reclamation, and rehabilitation: On the need for, and positing a definition of, ecological reclamation. *Restoration Ecology* 30(7):e13461. https://doi.org/10.1111/rec.13461

Gou S, Shibata S (2017) Assessing heritage trails: Trail conditions and influential managerial factors for the Nakahechi route on the Kumano Kodo pilgrimage network. *Landscape and Ecological Engineering* 13:251–263. https://doi.org/10.1007/s11355-016-0315-5

Hammitt WE, Cole DN, Monz CA (2015) Wildland recreation and resource impacts. In: *Wildland Recreation: Ecology and Management*, 3rd edn. John Wiley & Sons, Chichester, pp 3–14.

Hsieh H-F, Shannon SE (2005) Three approaches to qualitative content analysis. *Qualitative Health Research* 15(9):1277–1288. https://doi.org/10.1177/1049732305276687.

Hobbs RJ, Harris JA (2001) Restoration ecology: Repairing the Earth’s ecosystems in the new millennium. *Restoration Ecology* 9(2):239–246. https://doi.org/10.1046/j.1526-100X.2001.009002239.x

Hockett KS, Marion JL, Leung Y-F (2017) The efficacy of combined educational and site management actions in reducing off-trail hiking in an urban-proximate protected area. *Journal of Environmental Management* 203:17–28. <https://doi.org/10.1016/j.jenvman.2017.07.026>

International Mountain Bicycling Association (IMBA) (2004) *Trail solutions: IMBA’s guide to building sweet singletrack*. International Mountain Bicycling Association, Boulder, CO. ISBN 0975502301.

International Mountain Bicycling Association (IMBA) (2007) *Managing mountain biking: IMBA’s guide to providing great riding*. International Mountain Bicycling Association, Boulder, CO. ISBN 097550231X

International Mountain Bicycling Association (IMBA) (2025) *Construction*. <https://www.imba.com/construction>. Accessed 10 Aug 2025.

Kobayashi Y, Watanabe T (2023) Evaluation of the effectiveness of trail repair works based on three-dimensional monitoring around Mount Kurodake, Daisetsuzan National Park, Japan. *Sustainability* 15:12794. https://doi.org/10.3390/su151712794

Leung Y-F, Marion JL (1999) Assessing trail conditions in protected areas: Application of a problem assessment method in Great Smoky Mountains National Park, USA. *Environmental Conservation* 26(4):270–279. https://doi.org/10.1017/S0376892999000362

Levis C, Costa FRC, Bongers F, Peña-Claros M, Clement CR, Junqueira AB, Neves EG, et al. (2017) Persistent effects of pre-Columbian plant domestication on Amazonian forest composition. Science 355(6328):925–931. https://doi.org/10.1126/science.aal0157

Llena M, Batalla RJ, Vericat D (2024) Inferring on fluvial resilience from multi-temporal high-resolution topography and geomorphic unit diversity. *Geomorphology* 465:109412. <https://doi.org/10.1016/j.geomorph.2024.109412>

Llena M (2025) Can mountain bike-induced erosion be a major concern in natural areas? Evidence from high-resolution topography. *Science of the Total Environment* 1001:180532. <https://doi.org/10.1016/j.scitotenv.2025.180532>

Ma H, Wang Y, Yue H, Zhong B (2013) The threshold between natural recovery and the need for artificial restoration in degraded lands in Fujian Province, China. *Environmental Monitoring and Assessment* 185:8639–8648. https://doi.org/10.1007/s10661-013-3195-2

Marion JL (2016) A review and synthesis of recreation ecology research supporting carrying capacity and visitor use management decision making. *Journal of Forestry* 114(3):339–351. https://doi.org/10.5849/jof.15-062

Marion JL (2023) Trail sustainability: A state-of-knowledge review of trail impacts, influential factors, sustainability ratings, and planning and management guidance. *Journal of Environmental Management* 317:117868. https://doi.org/10.1016/j.jenvman.2022.117868

Marion JL, Wimpey J (2017) Assessing the influence of sustainable trail design and maintenance on soil loss. *Journal of Environmental Management* 189:46–57. <https://doi.org/10.1016/j.jenvman.2016.12.039>

Martins SV (2012) *Ecologia de Florestas Tropicais do Brasil*, 2nd edn. Editora UFV, Viçosa, MG. ISBN 978-85-7269-431-5.

Millennium Ecosystem Assessment (MEA) (2005) *Ecosystems and Human Well-Being: Synthesis*. Island Press, Washington, DC.

Mende P, Newsome D (2006) The assessment, monitoring and management of hiking trails: A case study from the Stirling Range National Park, Western Australia. *Conservation Science Western Australia* 5(3):285–295.

Miller AB, Blahna DJ, Morse WC, Leung Y-F, Rowland MM (2022) From recreation ecology to a recreation ecosystem: A framework accounting for social-ecological systems. *Journal of Outdoor Recreation and Tourism* 38:100455. <https://doi.org/10.1016/j.jort.2021.100455>

Moher D, Liberati A, Tetzlaff J, Altman DG, PRISMA Group (2009) Preferred reporting items for systematic reviews and meta-analyses: the PRISMA statement. *PLoS Medicine* 6(7):e1000097. <https://doi.org/10.1371/journal.pmed.1000097>

Monz CA, Pickering CM, Hadwen WL (2013) Recent advances in recreation ecology and the implications of different relationships between recreation use and ecological impacts. *Frontiers in Ecology and the Environment* 11(8):441–446. https://doi.org/10.1890/120358

Nelson CR, Hallett JG, Romero Montoya AE, Andrade A, Besacier C, Boerger V, Bouazza K, Chazdon R, Cohen-Shacham E, Danano D, Diederichsen A, Fernandez Y, Gann GD, Gonzales EK, Gruca M, Guariguata MR, Gutierrez V, Hancock B, Innecken P, Katz SM, McCormick R, Moraes LFD, Murcia C, Nagabhatla N, Pouaty Nzembialela D, Rosado-May FJ, Shaw K, Swiderska K, Vasseur L, Venkataraman R, Walder B, Wang Z, Weidlich EWA (2024) *Standards of Practice to Guide Ecosystem Restoration – A Contribution to the United Nations Decade on Ecosystem Restoration 2021–2030*. FAO, SER, and IUCN CEM, Rome/Washington, DC/Gland. <https://doi.org/10.4060/cc9106en>

Ng SL, Leung Y-F, Cheung S-Y, Fang W (2017) Land degradation effects initiated by trail running events in an urban protected area of Hong Kong. *Land Degradation & Development* 29(3):422–432. https://doi.org/10.1002/ldr.2907

Ng SL (2022) Is surfacing a good choice for trail management? Evidence from four high-use hiking trails in Hong Kong. *Journal of Soil and Water Conservation* 77(6):600–608. https://doi.org/10.2489/jswc.2022.00143

Olive ND, Marion JL (2009) The influence of use-related, environmental, and managerial factors on soil loss from recreational trails. *Journal of Environmental Management* 90:1483–1493. <https://doi.org/10.1016/j.jenvman.2008.10.004>

Pickering CM, Byrne J (2014) The benefits of publishing systematic quantitative literature reviews for PhD candidates and other early-career researchers. *Higher Education Research & Development* 33(3):534–548. https://doi.org/10.1080/07294360.2013.841651

Ramos-Scharrón CE, Reale-Munroe K, Atkinson SC (2014) Quantification and modeling of foot trail surface erosion in a dry sub-tropical setting. *Earth Surface Processes and Landforms* 39(13):1764–1777. https://doi.org/10.1002/esp.3558

Randall M, Newsome D (2008) Assessment, evaluation and a comparison of planned and unplanned walk trails in coastal south-western Australia. *Conservation Science Western Australia* 7(1):19–34.

Roovers P, Bossuyt B, Gulinck H, Hermy M (2005) Vegetation recovery on closed paths in temperate deciduous forests. *Journal of Environmental Management* 74(3):273–281. <https://doi.org/10.1016/j.jenvman.2004.10.003>

Salesa D, Cerdà A (2020) Soil erosion on mountain trails as a consequence of recreational activities: a comprehensive review of the scientific literature. *Journal of Environmental Management* 271:110990. https://doi.org/10.1016/j.jenvman.2020.110990

Schneller AJ, Binzen GL, Cameron C, Vogel ST, Bardin I (2020) Managing recreation in New York’s Adirondack Park: A case study of public perceptions and preferences for reducing user impacts to the High Peaks Wilderness Complex. *Journal of Park and Recreation Administration* 39(4):1–19. https://doi.org/10.18666/JPRA-2020-10523

Simão MVRC, Farías-Torbidoni EI, Llena M (2025) Management of degraded trails in protected natural areas worldwide: A systematic review of scientific literature. *Land Degradation & Development* 36:3279–3293. <https://doi.org/10.1002/ldr.5579>

Smith I, Pickering CM (2024) Assessing the environmental impacts, condition and sustainability of mountain biking trails in an urban national park. *Environmental Management*. <https://doi.org/10.1007/s00267-024-02029-6>

Snead JE, Erickson CL, Darling JA (2009) Making human space: The archaeology of trails, paths, and roads. In: Snead JE, Erickson CL, Darling JA (eds) *Landscapes of Movement: Trails, Paths, and Roads in Anthropological Perspective*. University of Pennsylvania Museum of Archaeology and Anthropology, Philadelphia, pp 1–19.

Sumanapala D, Wolf ID (2019) Recreational ecology: A review of research and gap analysis. *Environments* 6:81. https://doi.org/10.3390/environments6060081

Thurston E, Reader RJ (2001) Impacts of experimentally applied mountain biking and hiking on vegetation and soil of a deciduous forest. *Environmental Management* 27(3):397–409. https://doi.org/10.1007/s002670010157

Tomczyk AM, White PCL, Ewertowski MW (2016) Effects of extreme natural events on the provision of ecosystem services in a mountain environment: The importance of trail design in delivering system resilience and ecosystem service co-benefits. *Journal of Environmental Management* 166:156–167. <https://doi.org/10.1016/j.jenvman.2015.10.042>

**Supplementary Information (SI)**

| **Authors** | **Title** | **year** | **damage** | **impact** | **degradation** | **construction** | **build** | **repair** | **maintenance** | **maintain** | **recovery** | **rehabilitation** | **renaturalization** | **restoration** |
| --- | --- | --- | --- | --- | --- | --- | --- | --- | --- | --- | --- | --- | --- | --- |
| Aukerman, R. | The effectiveness of signing, pamphlets and restoration in reducing off-trampling | 1985 | 7 | 10 | 0 | 0 | 0 | 2 | 0 | 0 | 0 | 0 | 0 | 22 |
| Kuss, F. R.; Hall, C. N. | Ground flora trampling studies: Five years after closure | 1991 | 3 | 50 | 1 | 0 | 0 | 0 | 0 | 0 | 30 | 0 | 0 | 0 |
| Leung, Y-F | Assessing trail conditions in protected areas: application of a problem assessment method in Great Smoky Mountains National Park, USA | 1999 | 0 | 32 | 8 | 7 | 1 | 0 | 26 | 1 | 0 | 0 | 0 | 0 |
| Eagan, S.; Newman, P.; Fritzke, S.; Johnson, L. | Restoration of multiple-rut trails in the Tuolumne Meadows of Yosemite National Park | 2000 | 3 | 5 | 0 | 0 | 1 | 0 | 0 | 0 | 3 | 0 | 0 | 57 |
| Thurston, E.; Reader, R. J. | Impacts of experimentally applied mountain biking and hiking on vegetation and soil of a deciduous forest | 2001 | 16 | 43 | 4 | 0 | 0 | 0 | 0 | 0 | 3 | 0 | 0 | 0 |
| Roovers, P.; Bossuyt, B.; Gulinck, H.; Hermy, M. | Vegetation recovery on closed paths in temperate deciduous forests | 2005 | 1 | 5 | 0 | 0 | 0 | 0 | 0 | 0 | 44 | 0 | 0 | 3 |
| Mende, P.; Newsome, D. | The assessment, monitoring and management of hiking trails: A case study from the Stirling Range National Park, Western Australia | 2006 | 2 | 7 | 28 | 60 | 3 | 2 | 25 | 6 | 0 | 2 | 0 | 0 |
| Cahill, K. L.; Marion, J. L.; Lawson, S. R. | Exploring visitor acceptability for hardening trails to sustain visitation and minimise impacts | 2008 | 0 | 21 | 2 | 4 | 1 | 0 | 0 | 2 | 0 | 0 | 0 | 2 |
| Randall, M.; Newsome, D. | Assessment, evaluation and a comparison of planned and unplanned walk trails in coastal south-western Australia | 2008 | 0 | 1 | 23 | 2 | 0 | 0 | 50 | 0 | 0 | 1 | 0 | 0 |
| Grab, S.; Kalibbala, F. | ‘Anti-erosion’ logs across paths in the southern uKhahlamba–Drakensberg Transfrontier Park, South Africa: Cure or curse? | 2008 | 1 | 6 | 3 | 0 | 0 | 0 | 5 | 4 | 0 | 0 | 0 | 0 |
| Olive, Nathaniel D.; Marion, J. L. | The influence of use-related, environmental, and managerial factors on soil loss from recreational trails | 2009 | 1 | 25 | 7 | 12 | 0 | 0 | 14 | 15 | 0 | 0 | 0 | 0 |
| Aguirre, J. A. | Sustainable Trail Management in Costa Rica National Parks: The use of photography for trail surfacing decisions under tropical rainforest conditions | 2009 | 0 | 5 | 1 | 3 | 0 | 0 | 14 | 0 | 0 | 0 | 0 | 0 |
| Oh, Chi-Ok; Hammitt, W. E. | Determining Economic Benefits of Park Trails: Management Implications | 2010 | 0 | 7 | 1 | 9 | 0 | 2 | 24 | 4 | 0 | 0 | 0 | 0 |
| Buchwal, A.; Rogowski, M. | The methods of preventing trail erosion on the examples of intensively used footpaths in the Tatra and the Babia Góra national parks | 2010 | 11 | 9 | 0 | 3 | 0 | 2 | 20 | 2 | 0 | 0 | 0 | 0 |
| Ramos-Scharrón, C. E.; Reale-Munroe, K.; S. C. | Quantification and modeling of foot trail surface erosion in a dry sub-tropical setting | 2014 | 3 | 5 | 4 | 30 | 3 | 0 | 4 | 0 | 1 | 0 | 0 | 32 |
| Bie, K.; Vesk, P. A. | Ecological indicators for assessing management effectiveness: A case study of horse riding in an Alpine National Park | 2014 | 2 | 40 | 0 | 0 | 0 | 0 | 0 | 0 | 7 | 0 | 0 | 0 |
| Mason, S.; Newsome, D.; Moore, S.; Admiraal, R. | Recreational trampling negatively impacts vegetation structure of an Australian biodiversity hotspot | 2015 | 22 | 24 | 1 | 0 | 0 | 0 | 0 | 2 | 25 | 0 | 0 | 0 |
| Tomczyk, A. M.; White, P. C. L.; Ewertowski, M. W. | Effects of extreme natural events on the provision of ecosystem services in a mountain environment: The importance of trail design in delivering system resilience and ecosystem service co-benefits | 2016 | 24 | 30 | 1 | 13 | 2 | 26 | 8 | 2 | 15 | 5 | 0 | 20 |
| Marion, J. L. | A Review and Synthesis of Recreation Ecology Research Supporting Carrying Capacity and Visitor Use Management Decisionmaking | 2016 | 0 | 130 | 3 | 12 | 0 | 0 | 4 | 5 | 21 | 4 | 0 | 10 |
| Marion, J. L.; Wimpey, J. | Assessing the influence of sustainable trail design and maintenance on soil loss | 2017 | 1 | 11 | 14 | 12 | 1 | 0 | 25 | 10 | 0 | 0 | 0 | 0 |
| Hockett, K. S.; Marion, J. L.; Leung, Y-F | The efficacy of combined educational and site management actions in reducing off-trail hiking in an urban-proximate protected area | 2017 | 3 | 22 | 1 | 2 | 0 | 0 | 0 | 5 | 3 | 0 | 0 | 34 |
| Ng, S-L.; Leung Y‐F, Cheung S‐Y, Fang W. | Land degradation effects initiated by trail running events in an urban protected area of Hong Kong | 2017 | 3 | 44 | 33 | 1 | 1 | 3 | 4 | 1 | 23 | 0 | 0 | 0 |
|  |  |  |  |  |  |  |  |  |  |  |  |  |  |  |
| Gou, S.; Shibata, S. | Assessing heritage trails: trail conditions and influential managerial factors for the Nakahechi route on the Kumano Kodo pilgrimage network | 2017 | 4 | 16 | 5 | 32 | 4 | 2 | 26 | 9 | 0 | 2 | 0 | 10 |
| Kubo, T.; Shoji, Y.; Tsuge, T.; Kuriyama, K. | Voluntary Contributions to Hiking Trail Maintenance: Evidence From a Field Experiment in a National Park, Japan | 2018 | 0 | 1 | 0 | 0 | 0 | 0 | 19 | 1 | 0 | 0 | 0 | 0 |
| Drewnik, M.; Musielok, L.; Prędki, R.; Stolarczyk, M.; Szymański, W. | Degradation and renaturalization of soils affected by tourist activity in the Bieszczady Mountains (South East Poland) | 2019 | 2 | 10 | 24 | 0 | 0 | 0 | 0 | 0 | 0 | 0 | 44 | 5 |
| Schneller, A. J.; Binzen, G. L.; Cameron, C.; Vogel, S. T.; Bardin, I. | Managing Recreation in New York's Adirondack Park: A Case Study of Public Perceptions and Preferences for Reducing User Impacts to the High Peaks Wilderness Complex | 2020 | 10 | 22 | 8 | 2 | 4 | 0 | 3 | 2 | 5 | 0 | 0 | 2 |
| Ribet, S.; Brander, L. M. | Willingness to pay of trail runners for sustainable country park use in Hong Kong | 2020 | 0 | 11 | 7 | 0 | 0 | 0 | 13 | 0 | 0 | 0 | 0 | 0 |
| Ng, S. L. | Is surfacing a good choice for trail management? Evidence from four high-use hiking trails in Hong Kong | 2022 | 0 | 4 | 20 | 5 | 1 | 0 | 3 | 0 | 0 | 0 | 0 | 0 |
| Marion, J. L. | Trail sustainability: A state-of-knowledge review of trail impacts, influential factors, sustainability ratings, and planning and management guidance | 2023 | 2 | 60 | 27 | 21 | 1 | 1 | 25 | 12 | 2 | 0 | 0 | 1 |
| Kobayashi, Y.; Watanabe, T. | Evaluation of the Effectiveness of Trail Repair Works Based on Three-Dimensional Monitoring around Mount Kurodake, Daisetsuzan National Park, Japan | 2023 | 2 | 7 | 5 | 6 | 0 | 135 | 7 | 2 | 1 | 1 | 0 | 0 |
| Prevalence (%) |  |  | 21 | 30 | 24 | 19 | 12 | 9 | 20 | 18 | 14 | 6 | 1 | 12 |
| Total Ocurrences (n) |  |  | 123 | 663 | 231 | 236 | 23 | 175 | 319 | 85 | 183 | 15 | 44 | 198 |

**Suppementary Table S 1** Table presenting the 30 articles selected in the systematic review and the number of times each term appears in each article. At the end of the table, prevalence (percentage of articles in which each term) and total occurrences.

**References Cited in the Supplementary Information**

Aguirre JA (2009) Sustainable trail management in Costa Rica National Park: The use of photography in trail surface decisions under tropical rainforest conditions. *PASOS. Revista de Turismo y Patrimonio Cultural* 7(1):29–42.

Aukerman R (1985) The effectiveness of signing, pamphlets, and restoration in reducing off-trampling. *Tourism Recreation Research* 10(2):35–39. https://doi.org/10.1080/02508281.1985.11014573

Bie K, Vesk PA (2014) Ecological indicators for assessing management effectiveness: A case study of horse riding in an Alpine National Park. *Ecological Management & Restoration* 15(3):215–221. <https://doi.org/10.1111/emr.12122>

Buchwal A, Rogowski M (2010) The methods of preventing trail erosion on the examples of intensively used footpaths in the Tatra and the Babia Góra National Parks. *Geomorphologia Slovaca et Bohemica* 10(1):7–15.

Grab S, Kalibbala F (2008) ‘Anti-erosion’ logs across paths in the southern uKhahlamba–Drakensberg Transfrontier Park, South Africa: Cure or curse? *Catena* 73:134–145. <https://doi.org/10.1016/j.catena.2007.08.004>

Kubo T, Shoji Y, Tsuge T, Kuriyama K (2018) Voluntary contributions to hiking trail maintenance: Evidence from a field experiment in a national park, Japan. *Ecological Economics* 144:124–128. https://doi.org/10.1016/j.ecolecon.2017.08.022

Kuss FR, Hall CN (1991) Ground flora trampling studies: Five years after closure. *Environmental Management* 15:715–727. https://doi.org/10.1007/BF02589629

Mason S, Newsome D, Moore SA, Admiraal R (2015) Recreational trampling negatively impacts vegetation structure of an Australian biodiversity hotspot. *Biodiversity and Conservation* 24:2685–2707. https://doi.org/10.1007/s10531-015-0957-x

Oh CO, Hammitt WE (2010) Determining economic benefits of park trails: Management implications. *Journal of Park and Recreation Administration* 28(2):94–107.

Ribet S, Brander LM (2020) Willingness to pay of trail runners for sustainable country park use in Hong Kong. *Journal of Outdoor Recreation and Tourism* 31:100320. https://doi.org/10.1016/j.jort.2020.100320
